# A Cationic Porphyrin, ZnPor, Disassembles *Pseudomonas aeruginosa* Biofilm Matrix, Kills Cells Directly, and Enhances Antibiotic Activity of Tobramycin

**DOI:** 10.3390/antibiotics9120875

**Published:** 2020-12-06

**Authors:** Neha Patel, Shawn Swavey, Jayne Robinson

**Affiliations:** 1 Department of Biology, University of Dayton, Dayton, OH 45469, USA; pateln8@udayton.edu; 2 Department of Chemistry, University of Dayton, Dayton, OH 45469, USA; sswavey1@udayton.edu; 3 Integrated Science and Engineering Center, University of Dayton, Dayton, OH 45469, USA

**Keywords:** porphyrin, biofilm, *Pseudomonas aeruginosa*

## Abstract

One of the greatest threats to human health is the rise in antibiotic-resistant bacterial infections. *Pseudomonas aeruginosa* (PsA) is an “opportunistic” pathogen known to cause life-threatening infections in immunocompromised individuals and is the most common pathogen in adults with cystic fibrosis (CF). We report here a cationic zinc (II) porphyrin, ZnPor, that effectively kills planktonic and biofilm-associated cells of PsA. In standard tests against 16–18 h-old biofilms, concentrations as low as 16 µg/mL resulted in the extensive disruption and detachment of the matrix. The pre-treatment of biofilms for 30 min with ZnPor at minimum inhibitory concentration (MIC) levels (4 µg/mL) substantially enhanced the ability of tobramycin (Tobra) to kill biofilm-associated cells. We demonstrate the rapid uptake and accumulation of ZnPor in planktonic cells even in dedicated heme-uptake system mutants (ΔPhu, ΔHas, and the double mutant). Furthermore, uptake was unaffected by the ionophore carbonyl cyanide m-chlorophenyl hydrazine (CCCP). Cells pre-exposed to ZnPor took up the cell-impermeant dye SYTOX^TM^ Green in a concentration-dependent manner. The accumulation of ZnPor did not result in cell lysis, nor did the cells develop resistance. Taken together, these properties make ZnPor a promising candidate for treating multi-drug-resistant infections, including persistent, antibiotic-resistant biofilms.

## 1. Introduction

*Pseudomonas aeruginosa* (PsA) is a Gram-negative rod that causes infections in patients with compromised immunity [1,2,3]. This bacterium has been included in the top ten most dangerous antibiotic-resistant bacteria due to both intrinsic and acquired mechanisms of resistance [4,5,6]. The organism possesses many virulence factors (e.g., exoenzyme, exotoxin A, and elastase) [1,7] and forms complex communities known as biofilms: hydrated matrices of cells consisting of polysaccharides, extracellular DNA (eDNA), and proteins. PsA has been shown to form biofilms on abiotic (e.g., catheters and contact lenses) and biotic (e.g., urinary tract and lung tissue) surfaces [8,9,10], which are of significant medical importance, as they are more resistant to antibiotics than planktonic cells [1,11,12]. The antibiotic treatment of PsA has led to increased resistance to multiple antibiotics, creating multiple-drug-resistant (MDR) strains. Notably, PsA has developed resistance to many drugs, including antibiotics of the β-lactam, fluoroquinolone, and aminoglycoside families [13,14,15].

Given the high and rapidly increasing degree of bacterial resistance to traditional antibiotics, there is a great need for developing novel agents and mechanisms to treat these pathogens [16,17,18,19]. Indeed, a compound targeting and disrupting the biofilm matrix, subsequently rendering biofilm bacteria susceptible to antibiotics, would be of great value. One such class of compounds being explored are porphyrins. Porphyrins are a class of aromatic, heterocyclic compounds found in nature that play key roles in several organisms (e.g., heme, chlorophyll, cytochromes, etc.), yet they can be artificially synthesized to produce a high number of variants with different activities [20,21,22,23]. Interestingly, porphyrins are one of the earliest recognized classes of DNA ligands. Their interactions with dsDNA have long been studied and remain of great interest [24]. Studies have shown that many porphyrins, and their metal derivatives, have high affinity for DNA and bind by intercalation, external binding or aggregation, or both [25,26]. The extracellular matrix (ECM) of PsA biofilms contains high levels of eDNA, which may be a good target for porphyrin molecules [27,28,29]. The disruption of the matrix via porphyrin interactions with eDNA is likely to enhance accessibility to antibiotics and could even result in the detachment of the biofilm from the substrata. An added value in this scenario is that by disrupting eDNA, porphyrins may also disrupt horizontal gene transfer (HGT), an established mechanism that PsA and other bacteria use to pass antibiotic-resistance genes to other cells in the biofilm community [30,31,32,33].

In recent years, there has been increased interest in using the light activation of porphyrins to treat bacterial infections. This treatment is referred to as antimicrobial photodynamic therapy (aPDT), which is based on studies using the light activation of porphyrins to treat cancer via PDT [34,35,36,37,38,39,40,41,42,43,44]. Studies have shown the ability of cationic porphyrins to kill bacterial cells when photoactivated, via the production of reactive oxygen species (ROS); Gram-positive (*Staphylococcus aureus*) and Gram-negative (*Escherichia coli*) bacteria, as well as fungi (*Candida albicans*), are indeed susceptible to light-activated porphyrins [45,46,47,48,49]. Although the results of aPDT are promising using planktonic cells, less is known about their efficacy against biofilms. In addition, there are serious limitations in providing light to the sites of some of the most serious infections (e.g., PsA biofilm infections in the cystic fibrosis (CF) lungs). Such biofilms have a large surface area and are difficult to penetrate with light. Furthermore, the excitation wavelengths of most porphyrins are in the blue light range, which may cause tissue damage. Thus, efforts are underway to create porphyrins with excitation wavelengths in the infrared range to overcome this obstacle [50,51,52,53].

Previous studies in our laboratory have shown that a commercially available porphyrin, known as 5,10,15,20-tetrakis(1-methylpyridino)-21H,23H-porphine, tetra-p-tosylate salt (TMP), is highly effective against PsA biofilms and planktonic cells when photoactivated, while exhibiting little-to-no toxicity to bacterial cells in the absence of photoactivation [54]. However, we surprisingly discovered that when TMP interacted with PsA biofilms, it could cause disruption and detachment from the substrata without light activation. We also discovered that TMP could render the biofilm-associated cells of PsA sensitive to tobramycin, gentamycin, and vancomycin [54]; light activation was not required. To determine whether eDNA in the ECM was the target of TMP, we tested the effect of TMP on a wild-type PsA strain and a mutant (*pqsA*) strain that has little-to-no eDNA production in biofilms [54]. In these experiments, TMP was shown to have no effect on the *pqsA* mutant biofilms.

Herein, we sought to identify a porphyrin that could disrupt the biofilm matrix, as observed with TMP, and could directly kill bacteria without photoactivation. After screening numerous existing porphyrins, with none having the desired activities, our laboratory proceeded in constructing a porphyrin (Robinson and Swavey, 2014; US Patent # 9364537; 20). This porphyrin, which we designate as ZnPor (5,10,15-tris (*N*-methyl pyridyl)-20-pentafluorophenyl porphyrinatozincTris-4-methylbenzenesulfonate), is a cationic metallo-porphyrin with zinc as the central metal ion.

In this report, we propose the mechanism of action for ZnPor based on data indicating that ZnPor has direct antibacterial activity against PsA planktonic and biofilm-associated cells, as well as the ability to dissociate the biofilm matrix, in both the presence and absence of light.

## 2. Results

### 2.1. ZnPor Has a Direct Killing Effect on PsA Planktonic Cells

**Figure 1:**
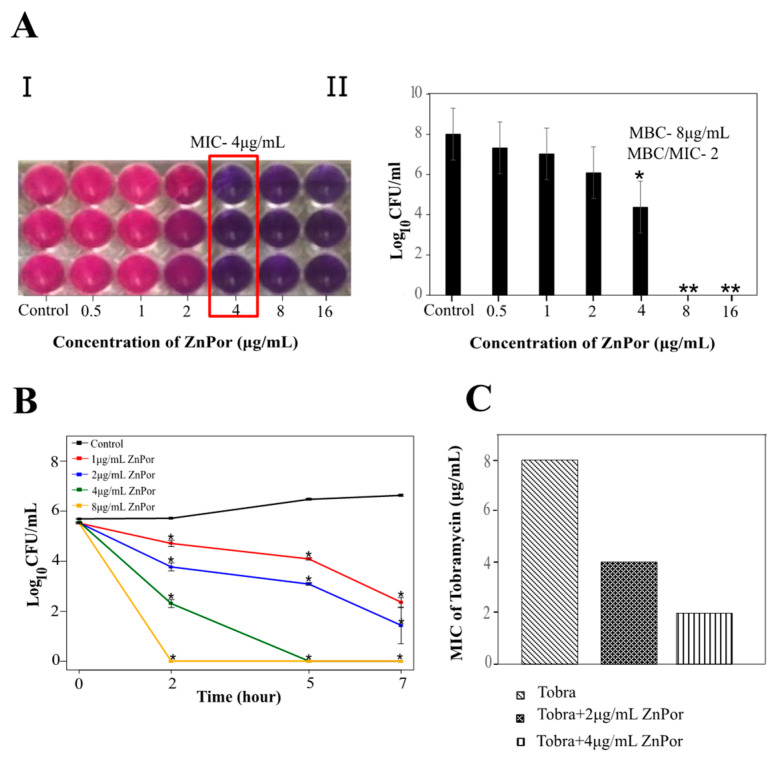
Antibacterial effect of ZnPor on planktonic *Pseudomonas aeruginosa* (PsA). (A.I.) and (A.II.) show minimum inhibitory concentration (MIC) and minimum bactericidal concentration (MBC) of ZnPor: Overnight PsA cultures were diluted to 10^4^ cells/mL. Mueller–Hilton broth was used as the testing medium as per Clinical and Laboratory Standard Institute (CLSI) guidelines, and minimum salts with 0.4% glucose were used as the testing medium for this study. (**A**) series of 2-fold dilutions of ZnPor was used to achieve concentrations in the wells from 0.5 to 16 µg/mL. (**I**) shows the reduction of the dye resazurin by live cells, from purple to pink. The lowest concentration (MIC) at which no growth was seen compared to control was indicated by the purple color. (**A.II**) represents viable plate counts of cell suspensions in the wells shown in (**A.I.**) MIC and the next two higher concentrations were plated on Luria Bertani (LB) to determine MBC. An MBC/MIC ratio <4 is considered a sign that an organism is susceptible to an antimicrobial. Additionally, we have yet to isolate any resistant variants. (**B**) Time–kill curve of planktonic PsA treated with ZnPor. Overnight PsA cultures were diluted to 10^4^ cells/mL in minimal salts and glucose (MSG) medium, and ZnPor was added at concentrations from 1 to 8 µg/mL. Controls received no ZnPor. Viable plate counts (CFU/mL) were determined by plating samples on LB agar at the time points shown. (**C**) Combinatory effect of Tobra and ZnPor on PsA planktonic cells. MIC of Tobra was compared to Tobra+ZnPor using the checkerboard micro-broth dilution method. All data points represent mean ± SD of three independent experiments (*n* = 9), with 3 samples per time point. * *p*-value < 0.005; ** no bacterial growth.

Our previous studies with TMP porphyrin demonstrated that it has little-to-no toxicity against planktonic PsA, except when irradiated with light [47]. ZnPor was surprisingly effective against planktonic PsA without light irradiation (Figure 1), which was not an effect observed with other porphyrins we had previously screened (data not shown). These experiments established ZnPor minimum inhibitory concentration (MIC; 4 µg/mL) and minimum bactericidal concentration (MBC) values (Figure 1A). A ratio of MBC/MIC less than 4 indicates a lack of resistance [55,56]. Notably, the MBC/MIC ratio for ZnPor against PsA was below 4 in all the experiments we conducted (Figure 1A). The time–kill curve for planktonic PsA shows 100% killing at a concentration of 8 µg/mL within 2 h of treatment (Figure 1B). Taken together, the evidence suggests that ZnPor could kill planktonic PsA.

### 2.2. ZnPor Destabilizes and Disrupts PsA Biofilms

**Figure 2:**
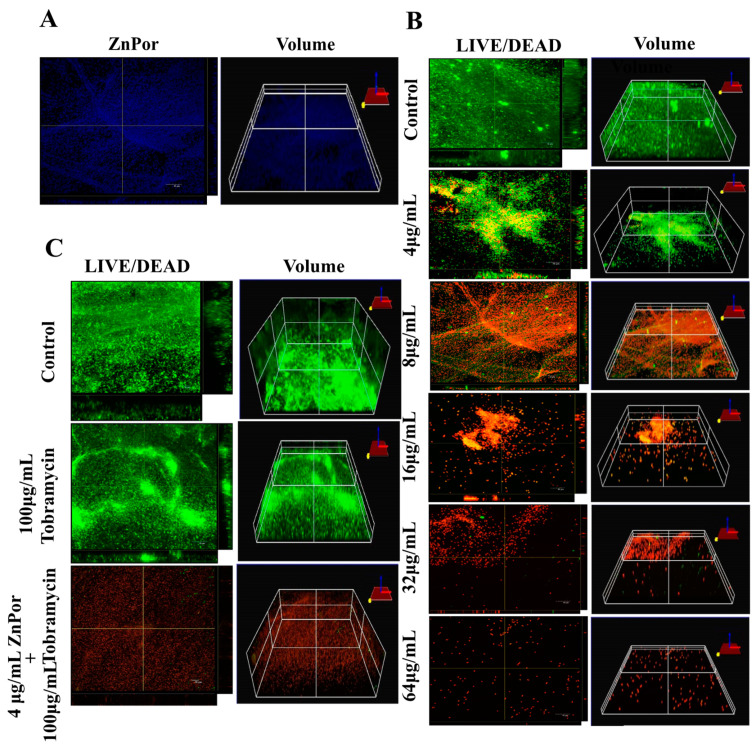
The effect of ZnPor on PsA biofilms: These experiments used confocal microscopy to image PsA biofilms formed on polyethylene coupons in Center for Disease Control (CDC) -approved bioreactors for 16–18 h. (**A**) ZnPor (alone)-stained biofilm. Biofilm was incubated in 8 µg/mL ZnPor solution for 2 h. The distribution of ZnPor in the biofilm was imaged by ZnPor excitation/emission (433/620 nm, respectively). The 3D volumetric depth image shows ZnPor was distributed throughout the biofilm matrix. (**B**) LIVE/DEAD^TM^ staining and 3D volumetric depths of biofilms treated with concentrations of ZnPor from 4 to 64 µg/mL for 2 h followed by LIVE/DEAD staining. (**C**) LIVE/DEAD^TM^ staining of biofilms treated with 100 µg/mL of Tobra alone, or in combination with ZnPor (30 min pre-treatment (4 µg/mL)), for 2 h. Biofilm treated with ZnPor alone (4 µg/mL) is shown in panel B. Control refers to a biofilm that received no treatment. All graphs are representative of three independent experiments (*n* = 9). Length of size bar: 10 µm.

The disruption of the biofilm matrix of PsA is highly desirable, regardless of where the biofilm forms, and this is especially the case for lung infections. Many antibiotics are not effective against biofilms because of their inability to penetrate the matrix [57,58]. As ZnPor (8 µg/mL) killed all the planktonic cells of PsA within 2 h of treatment, we tested the ability of ZnPor to penetrate the PsA biofilm matrix formed on polyethylene (PE) coupons after 16–18 h. Interestingly, ZnPor successfully penetrated the PsA biofilm matrix as shown by the fluorescence of ZnPor itself (Figure 2A). Furthermore, the killing effect of ZnPor on 18 h PsA biofilms was assessed using Confocal Laser Scanning Microscopy (CLSM) and LIVE/DEAD staining. In the absence of ZnPor, wild-type PsA cells formed dense biofilms on the polyethylene surfaces (PE), indicated by green cells (viable; Figure 2B). When wild-type PsA biofilms were exposed to different ZnPor concentrations, a concentration-dependent decrease in biofilm density was observed, and most cells within the biofilm were nonviable (Figure 2B).

### 2.3. The Effect of Tobra on Planktonic PsA Cells and ZnPor-Treated Biofilms

In this experiment, planktonic PsA cells, when treated with a combination of ZnPor (4 µg/mL) and Tobra, exhibited a decrease in the MIC of Tobra from 8 to 2 µg/mL, whereas sub-MIC levels of ZnPor resulted in a 2-fold decrease in the MIC of Tobra (Figure 1C). For biofilms, wild-type PsA biofilms were exposed to MIC levels (4 µg/mL) of ZnPor for 30 min, followed by exposure to 100 µg/mL of Tobra for 2 h. All the steps were performed in the dark. In the biofilms treated with Tobra, there was not a significant reduction in biofilm density and cell viability within the biofilms (Figure 2C). However, treatment with ZnPor and subsequent exposure to Tobra resulted in a substantial clearance of the biofilms and greater loss of cell viability throughout the biofilms than with either single treatment alone.

### 2.4. Localization of ZnPor in PsA Cells

**Figure 3:**
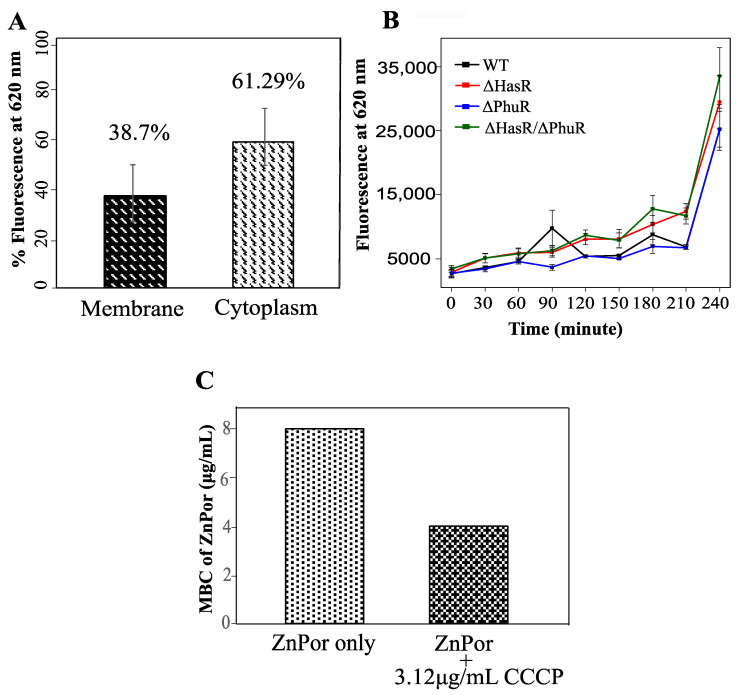
Uptake and distribution of ZnPor in PsA. (**A**) Levels of ZnPor in PsA cytoplasm of wild-type cells. Overnight PsA cultures were diluted to 0.15 optical density (OD) with MSG and exposed to 32 µg/mL ZnPor for 2 h. Cell suspensions were then sonicated (20 min) and centrifuged at 20,000× *g* for 30 min to separate cytoplasmic and membrane fractions. The level of fluorescence due to ZnPor in each fraction was measured. The percentage of ZnPor partitioned into the cytoplasm (67.3%) vs. cell wall (38.7%) fraction. (**B**) ZnPor uptake by PsA vs. ΔHasR, ΔPhuR, and ΔHasR/ΔPhuR mutants. All strains were grown in MSG medium containing 32 µg/mL ZnPor for 4 h. Fluorescence was measured (620 nm) at various time intervals (*x*-axis). (**C**) Minimum bactericidal concentration (MBC) of ZnPor against PsA wild-type planktonic cells treated with ZnPor vs. ZnPor in combination with a non-toxic concentration of the ionophore carbonyl cyanide m-chlorophenyl hydrazine (CCCP) (3.12 µg/mL). All data points represent mean ± SD of three independent experiments (*n* = 9).

The penetration and localization of ZnPor in the biofilm matrix led to the further investigation of the ZnPor distribution in the individual cells. After the treatment of PsA cells with ZnPor for 3 h, ZnPor accumulated more in the cytoplasm than in the membrane (Figure 3A). This indicates that the bactericidal mechanism of action not only contributed by making the cells permeable but also by interacting with the cytoplasmic material. This further implies the interaction of ZnPor with other biomolecules such as DNA, protein(s), etc.

### 2.5. ZnPor Uptake Is Not Dependent on the Dedicated Heme Uptake Systems of PsA

Many Gram-negative bacteria including the opportunistic pathogen PsA require iron for survival and virulence and therefore encode systems that utilize host heme-containing proteins as a source [1,2,3,4,5,6]. In fact, PsA encodes *phu* (Phu), a heme-uptake system, and *has* (Has), a heme-assimilation system [59]. Since ZnPor and heme share a similar structure, we investigated whether the dedicated heme uptake system of PsA (Phu/Has) is required in the uptake or assimilation of ZnPor. We used the ZnPor uptake assay to compare the ability of PsA-WT, ΔHasR, ΔPhuR, and ΔHasR/ΔPhuR PsA to take up ZnPor. Over 3.5 h, no significant difference was observed between PsA-WT and the mutants in ZnPor uptake (Figure 3B). This indicates that the uptake or transport of ZnPor in PsA cells is independent of the heme-uptake or assimilation systems.

### 2.6. Membrane Potential Is Not Required for ZnPor Uptake

Because heme pathways were not involved in the ZnPor uptake into PsA cells, we tested whether the uptake of ZnPor was due to changes in the membrane potential, or energy-mediated processes. Carbonyl cyanide m-chloro phenyl hydrazine (CCCP) is an ionophore that inhibits the membrane potential in bacterial cells by dissipating the proton motive force (PMF) [60,61]. Therefore, the addition of CCCP to ZnPor would inform as to whether membrane potential was required for ZnPor uptake by PsA. Interestingly, PsA cells treated with a nontoxic concentration of CCCP added to ZnPor [62] showed a 2-fold decrease in MBC as compared to cells treated with ZnPor alone (Figure 3C). Thus, the data suggest that ZnPor uptake does not require a change in membrane potential.

### 2.7. Cell Death from ZnPor Does Not Result in Cell Lysis

**Figure 4:**
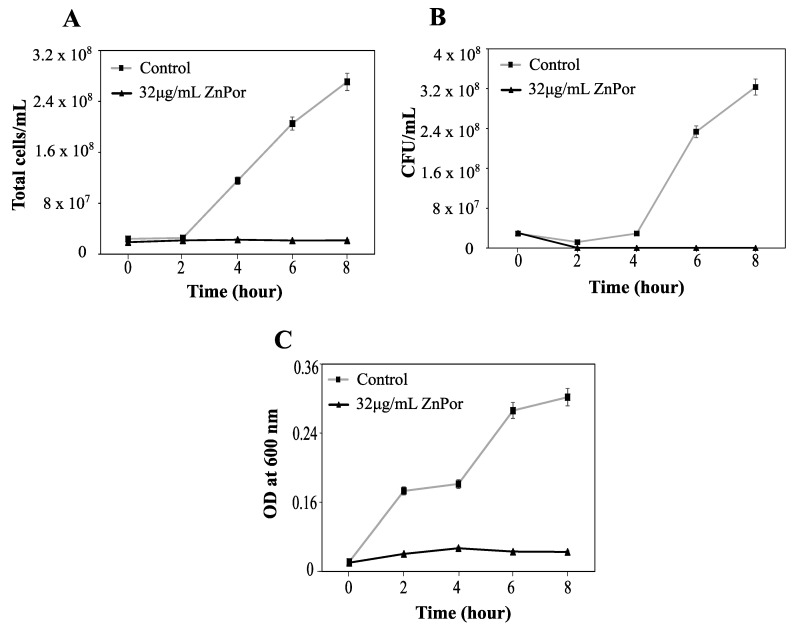
Viability and integrity of PsA after ZnPor treatment. PsA grown overnight in MSG were diluted in fresh MSG to 0.15 OD. ZnPor was added at a concentration of 32 µg/mL; control was without ZnPor. Samples were measured at various time intervals (*x*-axis). PsA cells were washed to remove extracellular ZnPor. The same cell suspensions were sampled for all three measurements. (**A**) Total cell counts (cells/mL) were calculated (Petroff–Hausser cell-counting chamber). (**B**) Viable cell counts (CFU per mL) were determined by plating the same cells on LB agar medium. (**C**) Cell suspensions (from **A**,**B**) were measured (at 600 nm) to determine whether cells had lysed or remained intact. All data points represent mean ± SD of three independent experiments (*n* = 9).

Many antibiotics that kill bacterial cells result in cell lysis, which causes the release of inflammatory cytoplasmic material and virulence factors. When PsA was treated with ZnPor, the bacteria remained intact (Figure 4A), indicating that ZnPor did not cause cell lysis. Had the cells lysed, there would have been a corresponding drop in absorbance at 600 nm. Instead, the absorbance at 600 nm was unchanged over a period of 3 h, while samples plated for viable counts showed a drop in viable cells over the same period. In addition, it was shown that the intact cells were non-viable after 3 h (Figure 4B). These non-viable cells stopped multiplying as compared to the controls (Figure 4C). Taken together, these data indicate that ZnPor can render the cells non-viable but does not lyse them.

### 2.8. ZnPor Makes PsA Cells Permeable

**Figure 5:**
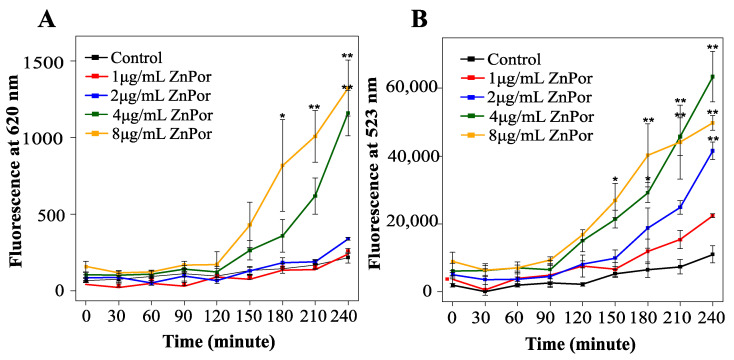
ZnPor effect on PsA permeability: PsA was grown overnight in MSG at 37 °C, diluted to 0.15 OD with fresh MSG, and subsequently treated with ZnPor at concentrations of 1 to 8 µg/mL (incubated at 37 °C at 180 rpm). Samples were removed at various time points and washed 2× with Tris buffer (pH 7.2) (**A**) ZnPor uptake: fluorescence analysis was completed (at 433 and 620 nm). (**B**) SYTOX^TM^ Green permeability assay: samples were treated with SYTOX^TM^ Green dye (10 min at 37 °C), and fluorescence (523 nm) was measured. Please note the lack of SYTOX^TM^ Green dye fluorescence in control cells not pre-treated with ZnPor. ZnPor at concentrations as low as 1 µg/mL resulted in permeabilization sufficient to allow the impermeable dye to enter and to bind to DNA. All data points represent mean ± SD of three independent experiments (*n* = 9). *p* values: * represents 0.05, and ** represents 0.002.

Given the findings that neither the heme uptake systems nor PMF are required for ZnPor transport and uptake, the ability of this compound to freely enter and permeabilize the PsA cell membrane was investigated. In that context, the uptake of ZnPor was measured followed by the uptake of the membrane-impermeable dye SYTOX^TM^ Green. Of note, ZnPor is a fluorescent molecule, which allows for its measurement by fluorescence uptake at 620 nm (Figure 5A). SYTOX^TM^ Green was chosen because it does not overlap with ZnPor fluorescence. SYTOX^TM^ Green is only able to enter cells when the cellular membranes are compromised or permeabilized. The addition of SYTOX^TM^ Green to ZnPor-treated cells (extracellular ZnPor was removed by repeated washes) showed an increased SYTOX^TM^ Green uptake that was dependent on the ZnPor concentration (Figure 5B). These results indicate that ZnPor makes PsA cells permeable.

### 2.9. Resistance against ZnPor

One of the major concerns in developing antimicrobials is the potential for bacteria to develop resistance. We observed no colony growth on the Luria Bertani (LB) plates from PsA cells incubated in 1×, 2×, 4×, and 8× MIC ZnPor concentrations, indicating no developed resistance against ZnPor.

## 3. Discussion

**Figure 6:**
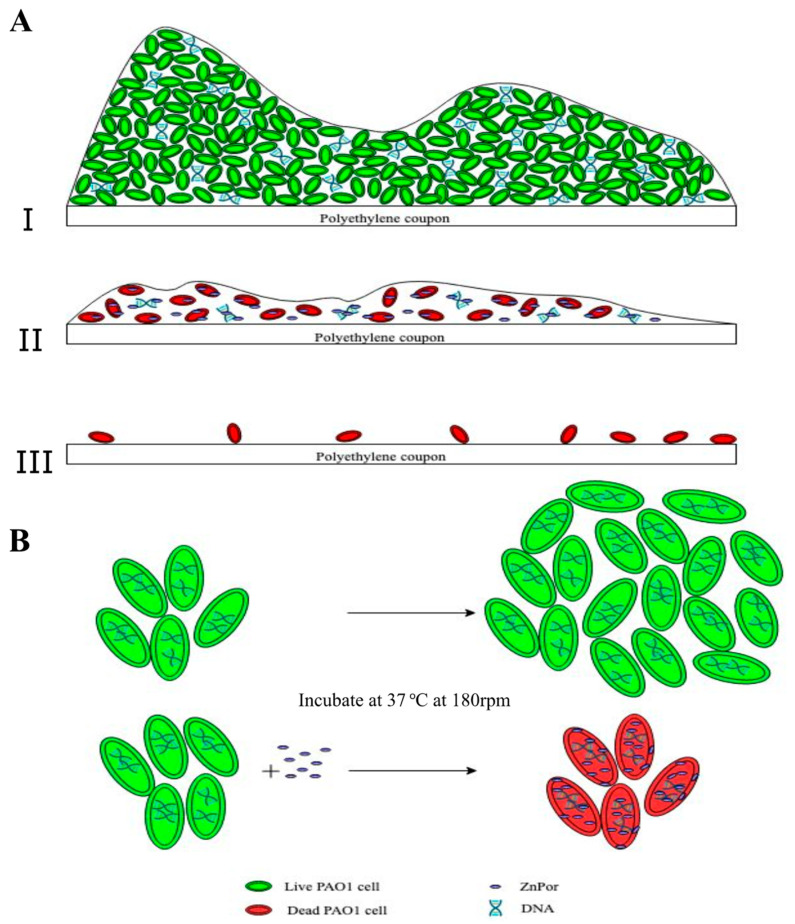
Proposed mechanism of action of ZnPor against PsA biofilms and individual cells. (**A.I**) The top panel represents a 16–18 h biofilm imaged after treatment with the LIVE/DEAD stain (as shown in Figure 2C control): a thick layer of cells encased in an extracellular matrix (ECM). All or almost all stained green with the LIVE/DEAD stain. The ECM contains a variety of different biomolecules, e.g., eDNA, which constitutes the majority of the ECM. ZnPor can diffuse throughout the ECM (as shown in Figure 2A) and thus interact with the eDNA in the matrix. (**A.II**) ZnPor treatment (prior to LIVE/DEAD) had the effect of destabilizing the biofilm, and ultimately, the biofilm sloughed off the surface (**II**,**III**). LIVE/DEAD-stained biofilms depict dead biofilm-associated cells left on the surface after ZnPor treatment. Overall, the matrices of biofilms treated with ZnPor were converted from thick and dense matrices to thin monolayers of almost exclusively dead cells, and the biofilms detached from the surfaces. (**B**) Individual planktonic PsA cells rapidly accumulated ZnPor in the cytoplasm and membrane/cell wall (Figure 3A). We hypothesize that ZnPor in the cytoplasm binds to the chromosomal DNA and inhibits replication. As shown in Figure 4A, ZnPor-treated cells did not increase in total number (nor did they decrease); these cells did not form colonies when plated onto LB agar plates and thus were not viable (Figure 4B). However, the cells did not lyse, as indicated in Figure 4C.

ZnPor is a cationic porphyrin with an antibacterial mechanism and the ability to deconstruct biofilm matrices of PsA. In this study, we explored the mechanism of this porphyrin in killing planktonic and biofilm-associated cells, as well as the mechanism involved in the deconstruction and detachment of the biofilm matrix. Therein, we propose a mechanism that explains what appears to be two independent activities; we postulate that DNA is a target in both activities (Figure 6).

Biofilms display a much higher resistance to killing by most antimicrobial compounds, up to 1000-fold greater, when compared to free-swimming planktonic cultures [12,13,63,64]. This is due to multiple factors, one being the biofilm extracellular matrix (ECM). The ECM is a thick layer composed of eDNA, polysaccharides, and proteins that is impervious to many compounds and, with its components being excellent anchors, keeps cells attached to a variety of animate and inanimate substrata [12,33,65,66,67]. Subsequently, antibiotics cannot reach the cells encased in the matrix. Biofilms are also a favorable environment for HGT via transformation, conjugation, and transduction [64,68,69]. ZnPor was created through a collaboration between the Robinson and Swavey laboratory groups at the University of Dayton [25,70]. Prior to the creation of ZnPor, we had screened a wide variety of porphyrins synthesized by S. Swavey, a known expert on porphyrins, as well as commercially available porphyrins. We found one, TMP, that effectively killed planktonic and biofilm-associated cells, when activated by light, qualifying it for use as an aPDT [54]. Notably, there was little-to-no toxicity against the individual control cells that were not exposed to light. This was expected, as all the porphyrins we tested and those reported in the literature did not possess “dark toxicity” towards bacterial cells. However, we noticed that TMP disrupted the biofilm matrix (in the absence of light activation) and rendered the biofilms more susceptible to Tobra and vancomycin [54]. ZnPor was then created using TMP as a template. In stark contrast, ZnPor exhibits strong antibiotic activity without light activation, yet it retains light-activated antimicrobial activity [34,45,46]. We further investigated the activity of this porphyrin by itself and in combination with Tobra, an aminoglycoside antibiotic, against overnight biofilms of PsA. Interestingly, we found that ZnPor was able to penetrate the entire biofilm and caused substantial disruption of the biofilm matrix, including detachment from the substrata (Figure 2A). Furthermore, the pre-treatment of biofilms with low concentrations of ZnPor (the MIC for planktonic cells) resulted in a substantial enhancement of killing by Tobra.

Our finding that ZnPor directly kills planktonic and biofilm-associated cells without photoactivation (Figure 6) was unexpected. To the best of our knowledge, there is no other porphyrin that can both directly kill planktonic and biofilm-associated cells, and disrupt the biofilm matrix, without requiring photoactivation. Bacterial resistance against porphyrins in general has not been previously reported. In line with previous reports, our own resistance testing showed that PsA cells did not develop resistance towards ZnPor.

Moreover, cell permeability assays showed that ZnPor could directly enter the cells, without requiring either of the bacterium’s dedicated heme uptake systems or PMF. Additionally, ZnPor made the cell membrane permeable to the impermeable dye SYTOX Green^TM^, and it did so at concentrations below the MIC. This is an interesting and highly valuable finding; Gram-negative cells are impermeable to many molecules, so our finding is surprising, and it likely explains the enhanced effectiveness of extant antibiotics as well as the increased spectrum of antibiotics not usually effective against PsA. This property could also be useful in research applications.

Further biofilm imaging showed the distribution of ZnPor throughout the matrix. It was also shown that it was rapidly taken up by the cells, distributed between the membrane and the cytoplasm. Intriguingly, the majority of the ZnPor was in the cytoplasm, where it had the opportunity to interact with the cell’s chromosomal DNA. The cytoplasm of the planktonic cells and the matrix of the biofilm contain many biomolecules such as proteins, DNA, RNA, and lipids, which could be possible targets of ZnPor. Fiel and co-workers previously initiated studies of cationic porphyrins, such as H2T4, and they demonstrated that these amphiphilic, water-soluble systems have a natural affinity for a potentially important intracellular target, namely, the DNA [71,72]. In accordance with these and other studies, our studies previously conducted in our lab support the interaction of eDNA in the biofilm matrix and the porphyrin. PsA biofilm treatment with TMP rendered the matrix porous, while the treatment of *pqsA* mutant (with little-to-no eDNA in the matrix) biofilms showed no significant effects [54,66,73,74,75,76,77,78]. Previous studies on ZnPor have demonstrated its high binding affinity for calf thymus DNA with a binding constant of 100,000 M^−1^ and no toxicity in the lung cancer cell line A549 up to 130 µg/mL [25]. Taken together, these data support two distinct actions of ZnPor, both involving DNA. Individual cells are killed by the uptake of ZnPor. Since the majority of ZnPor makes it into the cytoplasm, it is highly likely that it would intercalate into the chromosomal DNA, stalling replication. This hypothesis is further supported by the cell lysis study wherein we show that ZnPor can kill the PsA cells without lysing the cells and therefore keeping them intact, which could be a result of stalled replication in the cells. Naturally, when an antibiotic is effective in a clinical setting, bacteria are cleared. The process by which bacteria die under these circumstances can be highly inflammatory [79]. This leads to the release of highly inflammatory products, such as lipopolysaccharides (LPS), lipoteichoic acid (LTA), and peptidoglycan, collectively referred to as pathogen-associated molecular patterns (PAMPs), which are detected by innate immune receptors on many cell types. Perhaps the most well-discussed instance of the antibiotic-induced exacerbation of inflammatory responses is in the case of Gram-negative bacterial sepsis [80]. The ability of ZnPor to kill the cells without lysing them is certainly an added value and quite unique. Thus, ZnPor could be a potential candidate for future studies and be used as an enhancer for the MDR drugs against *Pseudomonas.* Overall, we have shown here that ZnPor is able to penetrate the entire biofilm matrix, as well as freely enter the cells. Given its ability to bind to PsA DNA (according to circular dichroism tests; data not shown) and disrupt the biofilm, this agent has the potential to eradicate persistent infections.

## 4. Materials and Methods

### 4.1. Bacterial Strains, Growth Conditions, and Chemicals

The PsA wild-type (WT-PAO1) and mutant strains ΔHasR, ΔPhuR, and ΔHasR/ΔPhuR were obtained from Eb Pesci (East Carolina University School of Medicine, Greenville, NC, USA). The PsA WT strains were grown aerobically with shaking in minimal salts and glucose medium (MSG) (40 mM K_2_HPO_4_, 20 mM KH _2_PO_4_, 7.6 mM [NH_4_]_2_SO_4_, 0.2 mM MgSO_4_7H_2_O, 9.2 × 10^−3^ mM FeCl_3_6H_2_O, and 0.2% (wt./vol) glucose; adjusted to pH 7.0) at 37 °C [4,17]. Mutants were streaked on appropriate antibiotic-selective media and then grown in liquid MSG, the same as the WT. A LIVE/DEAD^TM^ BacLight^TM^ bacterial viability assay kit was obtained from Thermo Fisher Scientific. SYTOX^TM^ Green dye was obtained from Invitrogen. Carbonyl cyanide m-chlorophenyl hydrazine (CCCP) and tobramycin (Tobra) were obtained from Sigma Aldrich.

### 4.2. Zinc Porphryin (ZnPor)

5,10,15-tris (*N*-methyl pyridyl)-20-pentafluoro phenyl porphyrinatozincTris-4-methylbenzenesulfonate) (ZnPor), was invented by Dr. Shawn Swavey based upon our results with TMP (US Patent # 9364537). Once its activity was characterized, it was synthesized commercially by a proprietary chemical group under contract to the University of Dayton. The porphyrin is soluble in water and fluoresces when excited at 433/620 nm excitation/emission. wavelengths. A 1 mg/mL stock solution was prepared in dH_2_O and filter sterilized. This stock was prepared fresh every week from the powder, filter sterilized, and kept at 4 °C wrapped in foil.

### 4.3. MIC/MBC and Kill Curve Assay

To determine the minimum inhibitory concentration (MIC) and the minimum bactericidal concentration (MBC) of ZnPor, we followed the standard CLSI guidelines for antimicrobial testing using the microdilution method [81]. Briefly, 96-well plates containing ZnPor and PsA were incubated overnight at 37 °C under static conditions. For combination studies of ZnPor and Tobra against planktonic cells of PsA, the standard checkerboard microdilution assay was used [82,83]. Briefly, a 10 × 6 array of serial 2-fold dilutions of Tobra and ZnPor were mixed together in a 96-well microtiter plate such that each row (or column) contained a fixed amount of one agent and increasing amounts of the second agent. Overnight-grown PsA cultures, diluted to 10^4^ cells/mL, were added to all the wells. The control PsA cells received neither of the antimicrobial treatments. To determine the MIC, 20 µL of 0.15 mM resazurin dye was added to all the wells and incubated in the above-mentioned conditions for an additional 2 h. The reduction of the dye by viable cells changed the color from purple to pink. Therefore, the MIC was determined as the lowest concentration at which the color of the dye was purple, which indicates that the cells are not viable. For the combination MIC, the lowest concentration combination that reduced the MIC compared to the MIC of Tobra was considered. The MBC was calculated by plating out samples of the MIC and the next two higher concentrations onto Luria Bertani (LB) agar. The lowest concentration that resulted in no colony growth on LB was determined to be the MBC. The MIC and MBC were measured in both the cation-adjusted Muller–Hilton broth and in the MSG medium, which was the medium used in biofilm formation. Blood was not included in the Muller Hilton (MH) broth because it reacts with porphyrins. For the kill curve, after overnight incubation, the PsA cultured in MSG was diluted to 10^5^ cells/mL. The diluted culture was treated with various concentrations of ZnPor and incubated at 37 °C under shaking conditions. Samples were removed at 0, 2, 5, and 7 h time points and were plated on LB medium for viability counts, CFU/mL.

### 4.4. Biofilm Growth Conditions and Confocal Laser Microscopy

PsA strains were grown overnight in MSG at 37 °C with shaking at 50 rpm. The following day, the bacteria were diluted in fresh media to an OD_600nm_ of 0.15. A volume of 150 mL of the standardized culture was added to CDC-approved bioreactors with polyethylene coupons and incubated for 16–18 h at 37 °C under shear conditions (50 rpm). Overnight biofilms formed on the polyethylene coupons in the bioreactors were observed using confocal scanning laser microscopy. The coupons were rinsed in sterile MilliQ water and submerged in MSG supplemented with ZnPor (only), ZnPor + Tobra, or Tobra alone. The coupons were then stained with the LIVE/DEAD^TM^ BacLight bacterial viability dyes where noted (Molecular Probes Inc., Eugene, OR) containing SYTO9 (excitation at 480 nm and/or at 500 nm) and propidium iodide (excitation at 490 nm and emission at 635 nm) dyes. The negative control coupons were suspended in MSG only. For the combination treatment, biofilms were first treated with 4 µg/mL of ZnPor for 30 min, followed by treatment with Tobra (100 µg/mL), for a total time of 2 h. ZnPor is a naturally fluorescent molecule (excitation at 433 nm and emission at 620 nm). Thus, we could observe the distribution of ZnPor when it was used alone to stain biofilms. The biofilms were visualized with an Olympus FV1000 CLSM (Olympus America, Center Valley, PA, USA) using a 60× oil-immersion objective. Biofilm images were acquired in 1.5 µm optical sections for the entire thickness of the biofilm.

### 4.5. Uptake and Localization of ZnPor in PsA Cells

Overnight PsA cultures grown in MSG were diluted to a 0.15 OD_600nm_ with fresh MSG and subsequently treated with different concentrations of ZnPor and incubated at 37 °C under shaking conditions. For the ZnPor uptake assay, 1 mL samples were taken every 30 min. The samples were washed 2× with Tris buffer (pH 7.2), and fluorescence was measured at 620 nm to detect the uptake of ZnPor into the PsA cells. For the localization assay, the diluted overnight PsA culture was harvested and concentrated 30× in Tris buffer. The PsA cells were re-suspended in Tris buffer (pH 7.2) and vortexed until the cells were completely suspended. The PsA were lysed at 4 °C by sonication for 20 min (1 s on; 1 s off) using a Fisher^®^ 550 Sonic Dismembrator (with a Minonix incorporated convertor and a horn with a ½″ diameter tip). The sonicated cells were centrifuged 3× *g* for 30 min at 20,000× *g* and 4 °C in a Beckman Avanti^TM^J-25 centrifuge with the JA-25-50 rotor. The sonicated cells were separated into pellets and supernatants (cell-free lysates), and the relative amounts of ZnPor in each fraction were determined by measuring the ZnPor fluorescence.

### 4.6. Cell Lysis Assay

Overnight PsA cultures grown in MSG were diluted to 10^5^ cells/mL. This diluted culture was treated with 32 µg/mL of ZnPor at the MBC and incubated at 37 °C under shaking conditions for 8 h. The cells recovered from both the control and treated samples were centrifuged at 5000× *g* for 10 min and washed twice with MSG at various time intervals. The cells were observed under bright field and fluorescence microscopy using a 60× oil-immersion objective (Olympus America, Center Valley, PA, USA). The total counts of cells/mL were determined using a Petroff–Hausser bacterial cell counting chamber. Additionally, the optical density of the cell suspension at 600 nm was recorded at the same time. The viable cell counts (CFU per mL) were determined by plate counts on LB agar. All the experiments were conducted in the absence of light activation.

### 4.7. Effect of ZnPor on Membrane Permeability of PsA Cells

Overnight cell cultures grown in MSG were diluted to a 0.15 OD with fresh MSG. The cell suspension was aliquoted into separate tubes, treated with various concentrations of ZnPor, and incubated at 37 °C under shaking conditions. At 30 min intervals, 1 mL aliquots of the ZnPor-treated cell suspensions were removed, washed 2× with Tris buffer (pH 7.2) and transferred to 96-well plates, where they were then spiked with the cell-impermeant SYTOX^TM^ Green dye (excitation: 504 nm/emission: 523 nm) at a final concentration of 2.5 µM. After 10 min of incubation, the fluorescence due to the SYTOX^TM^ Green dye binding to DNA in the PsA cells was measured at 523 nm.

### 4.8. Resistance Assay

This assay was used to determine whether PsA developed resistance to ZnPor. Bacterial cultures (10^4^ cells/mL) in MSG were treated with control (no ZnPor) or ZnPor at 1, 2, 4, and 8× the MIC concentration under shaking conditions at 37 °C. The cultures were sampled every 24 h, diluted, and plated on LB agar plates (with and without ZnPor). Sampling and testing were repeated for 7 days. No colonies were observed on the LB plates treated with ZnPor. The only colonies observed on the LB plates were from the control (no ZnPor) and cells incubated at the MIC of ZnPor. These findings indicate the absence of resistance in PsA against ZnPor.

## 5. Conclusions

ZnPor exhibits antibacterial activity against both the planktonic and biofilm-associated cells of PsA. In addition to killing the cells directly, ZnPor interacts with the biofilm, resulting in the dismantling of the matrix. This allows extant antibiotics such as tobramycin that are ineffective against biofilms to regain their activity. None of this activity requires the light activation commonly used in PDT. Additionally, ZnPor at sub-MIC levels is able to make the cells permeable to other molecules and, specifically, to restore sensitivity in MDR strains of PsA.
